# Small Fibre Peripheral Alterations Following COVID-19 Detected by Corneal Confocal Microscopy

**DOI:** 10.3390/jpm12040563

**Published:** 2022-04-01

**Authors:** Edoardo Midena, Eleonora Cosmo, Anna Maria Cattelan, Chiara Briani, Davide Leoni, Alfio Capizzi, Vanessa Tabacchi, Raffaele Parrozzani, Giulia Midena, Luisa Frizziero

**Affiliations:** 1 Ophthalmology Unit, Department of Neuroscience, University of Padova, 35128 Padova, Italy; vanessa.tabacchi@studenti.unipd.it (V.T.); raffaele.parrozzani@unipd.it (R.P.); luisa.frizziero@unipd.it (L.F.); 2 IRCCS—Fondazione Bietti, 00198 Rome, Italy; eleonora.cosmo@fondazionebietti.it (E.C.); giulia.midena@fondazionebietti.it (G.M.); 3 Unit of Infectious Disease, Department of Internal Medicine, University of Padova, 35128 Padova, Italy; annamaria.cattelan@aopd.veneto.it (A.M.C.); davide.leoni@aopd.veneto.it (D.L.); 4 Neurology Unit, Department of Neuroscience, University of Padova, 35128 Padova, Italy; chiara.briani@unipd.it; 5 Department of Directional Hospital Management, University of Padova, 35128 Padova, Italy; alfio.capizzi@aopd.veneto.it

**Keywords:** corneal confocal microscopy, COVID-19, small fiber, neuropathy, cornea, SARS-CoV-2

## Abstract

A large spectrum of neurological manifestations has been associated with coronavirus disease 2019 (COVID-19), and recently, the involvement of small fibers has been suggested. This study aims to investigate the involvement of small peripheral nervous fibers in recovered COVID-19 patients using in-vivo corneal confocal microscopy (CCM). Patients recovered from COVID-19 and a control group of healthy subjects underwent in-vivo CCM. Corneal nerve fiber density (CNFD), corneal nerve branch density (CNBD), corneal nerve fiber length (CNFL), corneal nerve fiber total branch density (CTBD), corneal nerve fiber area (CNFA), corneal nerve fiber width (CNFW), fiber tortuosity (FT), number of beadings (NBe), and dendritic cells (DC) density were quantified. We enrolled 302 eyes of 151 patients. CNBD and FT were significantly higher (*p* = 0.0131, *p* < 0.0001), whereas CNFW and NBe were significantly lower (*p* = 0.0056, *p* = 0.0045) in the COVID-19 group compared to controls. Only CNBD and FT resulted significantly correlated to antiviral drugs (increased) and corticosteroids (decreased). No significant relationship with disease severity parameters was found. COVID-19 may induce peripheral neuropathy in small fibers even months after recovery, regardless of systemic conditions and therapy, and CCM may be a useful tool to identify and monitor these morphological changes.

## 1. Introduction

Coronavirus disease 2019 (COVID-19) has become a global pandemic caused by the highly transmissible severe acute respiratory syndrome coronavirus 2 (SARS-CoV-2). This beta-coronavirus is responsible for a high morbidity burden in affected patients, because it may cause permanent damage to the tissues involved during the acute phase. Neurological involvement plays a significant role in the clinical manifestations of this disease, both in the acute phase and in long-term recovery [1]. A large spectrum of neurological manifestations has been associated with the infection, involving both central and peripheral nervous systems, including polyradiculoneuritis (Guillain–Barre syndrome), meningitis, encephalomyelitis, and encephalopathy [2,3].

Recently, the involvement of small fibers has been suggested in COVID-19, the confirmation of which would be of great relevance both for its pathophysiological implications in the virus-related mechanisms of damage and for the relevant burden that small-fiber neuropathy (SFN) may cause in the affected patients [4,5,6].

Ocular involvement in COVID-19 has already been described. SARS-CoV-2 RNA was detected in conjunctival swabs from infected patients and the most frequent and early ocular sign of COVID-19 is keratoconjunctivitis [7]. The cornea is the most densely innervated structure in the human body [8]. The damage of the corneal sub-basal nerve fibers has been well described in patients affected by SFN of different causes [5,9,10,11,12]. Besides clinical evaluation, the diagnosis and follow-up of peripheral neuropathy may require invasive examinations, such as skin biopsies [5,11]. In-vivo corneal confocal microscopy (CCM) is a non-invasive imaging technique for the study of corneal cellular structure, particularly the corneal nervous plexuses, providing images which are comparable to ex vivo histochemical techniques and useful in the early diagnosis of SFN, detecting early and sometimes subclinical changes [5,12].

This study aimed at investigating the potential impact of COVID-19 on small peripheral corneal nerve fibers using CCM, a non-invasive in vivo diagnostic tool, in patients recovered from COVID-19.

## 2. Materials and Methods

### 2.1. Study Population

In this observational cross-sectional study, patients recovered from COVID-19 and followed at the Unit of Infectious Diseases of Padova University Hospital were consecutively recruited between November 2020 and January 2021. All the approached subjects consented to participate in the study. COVID-19 infection was initially detected and then monitored through a molecular reverse transcription-polymerase chain reaction (RT-PCR) test. A group of age-matched subjects, free of any systemic or ocular disease, and/or assuming any topical or systemic therapy, who voluntarily chose to be enrolled in this study, served as the control group. None of the control group subjects reported a positive history of COVID-19 infection confirmed by oropharyngeal swabs, nor experienced any symptoms related to COVID-19 infection, and presented a negative RT-PCR test within one month before enrolment. No study participant was vaccinated against COVID-19. Institutional ethics committee approval was obtained, and all subjects gave their informed consent before enrollment in the study, in accordance with the tenets of the Declaration of Helsinki.

Inclusion criteria for the patient group were: age ≥ 18 years, previous COVID-19 infection confirmed by two consecutive oropharyngeal swabs positive for the SARS-CoV-2 genome. Recovery was confirmed by two consecutive negative swabs, resolution of symptoms, and detection of anti-SARS-CoV-2 IgGs in blood samples. Exclusion criteria for both groups were: history of any ocular discomfort or ophthalmologic disease that may affect the normal morphology of the cornea, including history of significant bacterial, fungal, or viral keratitis, history of ocular surgery, trauma, or foreign bodies, recurrent ulcerations, corneal ectasias, dystrophies or degenerations, corneal congenital anomalies, and use of contact lenses in the last three weeks; any ocular or systemic disease, including diabetes, which might cause peripheral neuropathy, including corneal neuropathy. Patients requiring any therapy for ocular diseases, including artificial tears and lubricants for dry eye, were also excluded. Patient records were reviewed for other systemic comorbidities, such as hypertension, cardiovascular and cerebrovascular events, and previous or ongoing chemotherapy, which were taken into account as potential confounders in planned analysis. Moreover, for each subject of the COVID-19 group, the following data related to the disease course and management were recorded: time of positivity (from the first positive SARS-CoV-2 test to the first negative SARS-CoV-2 test), hospitalization period, admission to intensive care, need for oxygen therapy, mechanical ventilation, intubation and pharmacological therapy, including corticosteroids (namely prednisolone, prednisone, methylprednisolone, betamethasone), non-steroidal anti-inflammatory drugs (NSAID, namely, ketoprofen lysine salt, diclofenac, ketorolac tromethamine), paracetamol, antibiotics (namely, azithromycin, amoxicillin and clavulanate, levofloxacin, piperacillin and tazobactam, doxycycline, ceftriaxone, meropenem, linezolid, vancomycin, teicoplanin, clarithromycin), antiviral drugs (namely, lopinavir/ritonavir, remdesivir), hydroxychloroquine and/or chloroquine, heparin and/or other anticoagulants, biological drugs (namely tocilizumab), plasma, and glutathione.

Finally, all clinical manifestations and symptoms experienced from the beginning of the disease were investigated and recorded for each enrolled patient: fever, gastrointestinal symptoms (i.e., nausea, vomiting, abdominal pain, diarrhea), respiratory symptoms (i.e., dyspnea, shortness of breath, dry cough), osteoarticular symptoms (i.e., bone and osteoarticular pain, myalgia), ageusia/anosmia, perception of visual acuity impairment, ocular pain, redness and/or burning eye sensation, dizziness, headache, syncope/fainting, language disorders, cognitive impairment, and memory disorders.

### 2.2. Corneal Confocal Microscopy

All enrolled subjects underwent CCM, using the Heidelberg Retina Tomography with the Rostock Cornea Module (HRTIII/RCM, Heidelberg Engineering, Heidelberg, Germany). Both eyes of each subject were evaluated. The HRTIII employs a 670 nm wavelength diode laser source and provides cross-sectional images of 400 × 400 µm with a lateral resolution of 1 µm. For CCM imaging, a disposable sterile polymethylmethacrylate cap (TomoCap; Heidelberg Engineering, Heidelberg, Germany) filled with hydroxypropyl methylcellulose 2.5% (GenTeal gel; Novartis Ophthalmics, East Hanover, NJ, USA) was placed on the objective lens of the cornea module to improve optical coupling. After the instillation of topical anaesthesia, the corneal module was advanced manually until obtaining an appropriate cap contact with the corneal surface. Using the section mode of the CCM, layer per layer images of the full thickness cornea were obtained according to our standard acquisition protocol. The operator started to acquire images from the corneal epithelium, and then manually shifting the focus through the cornea, images of the sub-basal plexus, of the anterior middle and posterior stroma, and of the endothelium were captured as well. Approximately 300 images were obtained per eye. For the aim of this study, we then evaluated only the sub-basal nerve plexus. Images representing the inferior whorl and the peripheral cornea were excluded from further analyses. The three best focused, non-overlapping images of the sub-basal nerve plexus were selected for each of the examined eyes according to a previously accepted method, and the average of the derived measures was used for further analyses [13,14,15,16]. Firstly, a quantitative automated image analysis software (ACCMetrics, software version 2.0, 03-2013; University of Manchester, Manchester, UK, courtesy of Prof. Rayaz A. Malik) was used to calculate six parameters: corneal nerve fiber density (CNFD), the number of nerve fibers/mm^2^; corneal nerve branch density (CNBD), the number of primary branch points on the main nerve fibers/mm^2^; corneal nerve fiber length (CNFL), the total length of nerves mm/mm^2^; corneal nerve fiber total branch density (CTBD), the total number of branch points/mm^2^; corneal nerve fiber area (CNFA), the total nerve fiber area mm^2^/mm^2^; corneal nerve fiber width (CNFW), the average nerve fiber width mm/mm^2^ [17,18,19].

Two other relevant parameters were obtained by a manual quantitative analysis performed by a blinded, experienced operator on the best-quality image of the sub-basal nerve plexus, namely the number of beadings (NBe) and fiber tortuosity (FT), according to previously reported methods [9]. NBe was defined as the number of hyperreflective points per unit of length (100 μm) of the best focused fiber, randomly chosen by the operator from all the nerve fibers seen in the corneal sub-basal nerve plexus image. Nerve beadings represent the accumulation of mitochondria along the nerve, thus documenting the metabolic activity of corneal fibers of the sub-basal nerve plexus [9,13]. FT was classified using the grading system proposed by Oliveira-Soto, which simultaneously considers the frequency and amplitude of changes in nerve fiber direction and provides a score ranging from 0 to 4, where 0 represents almost straight nerve fibers, 1 slightly tortuous fibers, 2 moderately tortuous fibers, 3 tortuous fibers with a quite severe amplitude of changes in fiber direction, and 4 very tortuous nerve fibers with abrupt and frequent changes in direction [20]. FT is considered a morphologic marker of nerve degeneration and an attempt to fiber repair [9,21].

Finally, using the same images, we quantified dendritic cell (DC) density. One expert operator manually counted the highly reflective cellular structure with and without a branching dendritic morphology, thus considering both the mature and immature population of DCs, respectively [22,23]. DC density was then calculated as the number of cells divided for the area of the captured image expressed in mm^2^ (cells/mm^2^).

All exams were conducted by a blinded examiner, and all image analyses were performed randomly on anonymized images by blinded operators.

### 2.3. Statistical Analysis

All variables were summarized according to the usual methods of descriptive statistics: mean and standard deviation for quantitative variables; absolute and relative (percentage) frequencies for qualitative variables.

To obtain the sample size, the comparison between the COVID-19 group and control group has been performed using a two-sided test with 95% confidence level (type I error alpha = 0.05) and power 1-beta = 0.80. Sample size was determined first in terms of the number of patients and controls, with an allocation ratio of 3:1, necessary to recognize the effect sizes of about 0.5 (medium size) as statistically significant. Because measures on both eyes of subjects were available, the total sample size of 170 subjects has been translated in terms of the number of eyes assuming an average correlation between the measures of the two eyes equal to 0.55. The number of 378 eyes thus obtained—the minimum sample size requested for the study—has been broken down into 284 eyes of 142 COVID-19 patients and 94 eyes of 47 control subjects.

Corneal sub-basal nerve plexus parameters (CNFD, CNBD, CNFL, CTBD, CNFA, CNFW, FT, and NBe) and DC density were compared between COVID-19 and control groups by means of the ANOVA model, adjusted for the replication of measures in both eyes of the same patient.

The relationship between corneal sub-basal nerve plexus parameters that resulted significantly different in the univariate analysis (CNBD, CTBD, CNFW, FT, and NBe) and the severity of the disease (need for hospitalization, and time of hospitalization, admission to intensive care unit, need for oxygen treatment) and treatments was analyzed by means of a multiple linear regression analysis, adjusted for some potential confounders (arterial hypertension, previous cardiovascular events, previous and/or current chemotherapy). Variables to be included in the multiple regression model were chosen among those showing a significant relationship (*p* < 0.10) in a previous univariate linear regression analysis. In all models, parameters were entered as dichotomic independent variables (yes vs. no), except for time of hospitalization, which entered the model as a continuous variable: the regression coefficient of this parameter was used to evaluate the statistical significance of its relationship with the dependent variable. As regards the adjustment of *p*-values for type I error inflation due to multiple testing, we believe Bonferroni’s correction to be too conservative to evaluate significant results in this research contest. Thus, we applied the control of false discovery rate (FDR) using the Benjamini–Hochberg procedure, which confirmed that all significant, not adjusted *p*-values we found could be interpreted as significant.

The correlation between CCM parameters and time since recovery was also studied using a regression model taking into account the replication of measures on both eyes of each patient (PROC MIXED with REPEATED statement).

Finally, the relationship between the significantly altered corneal sub-basal nerve plexus parameters in COVID-19 groups (CNBD, CTBD, CNFW, FT, and NBe) and symptoms was assessed by means of simple logistic regression. Data were analyzed using SAS^®^ 9.4 statistical software (SAS Institute, Cary, NC, USA). A value of *p* < 0.05 was considered statistically significant.

## 3. Results

A total of 302 eyes from 151 patients (83 men and 68 women, mean age of 56.8 ± 14.2 years) who recovered from COVID-19 were enrolled. The control group included 92 eyes from 46 (16 men, 30 women, mean age of 49.4 ± 26.5 years) healthy subjects. No significant age difference was found between patients and control group (*p* = 0.0762). Forty-seven patients (31.1%) had arterial hypertension, 11 (7.3%) a history of cardiovascular and/or cerebrovascular events, and one patient (0.7%) had previous chemotherapy treatment. COVID-19 patients were positive at the molecular COVID-19 test for a mean time of 19.3 ± 10.5 days, and all patients complained of symptoms, including: fever (137, 90.7%), gastrointestinal (55, 36.4%), respiratory (122, 80.8%),and osteoarticular symptoms (68, 45.0%), ageusia/anosmia (104, 68.9%), perception of reduction in visual acuity (35, 23.2%), ocular pain (8, 5.3%), redness and/or burning eye sensation (19, 12.6%), dizziness (36, 23.8%), headache (69, 45.7%), syncope/fainting (18, 11.9%), language disorders (20, 13.2%), cognitive impairment (56, 37.1%), and memory disorders (49, 32.5%). A total of 135 (89.4%) COVID-19 patients required hospitalization, whose mean duration was 13.0 ± 2.6 days. Thirty (22.2%) of the hospitalized patients needed intensive care unit management. Oxygen therapy was required in 90 (66.7%) hospitalized patients, 16 (11.9%) needed mechanical ventilation, and seven (5.2%) required intubation. All patients, except for three, underwent pharmacologic therapy with at least one of the following drugs: 85 (56.3%) received corticosteroids, five (3.3%) NSAID (one of them (20%) ketoprofen lysine salt, one (20%) diclofenac, three (60%) ketorolac tromethamine), 116 (76.8%) paracetamol, 125 (82.8%) antibiotics, 59 (39.1%) antiviral drugs (24 of them (40.7%) lopinavir/ritonavir, 33 (55.9%) remdesivir, and two with both of them (3.4%)), 63 (41.7%) hydroxychloroquine and/or chloroquine, 115 (76.2%) heparin and/or other anticoagulants, 10 (6.6%) biological drugs (i.e., tocilizumab), 21 (13.9%) plasma, and 17 (11.3%) glutathione. COVID-19 infection related symptoms reported by patients were resolved at the time of enrollment and none of the aforementioned therapies was still ongoing at the time of examination. Mean time from recovery (negative swab) was 144.4 ± 104 days (Table 1).

The analysis of CCM in COVID-19 and control groups is reported in Table 2.

**Figure 1:**
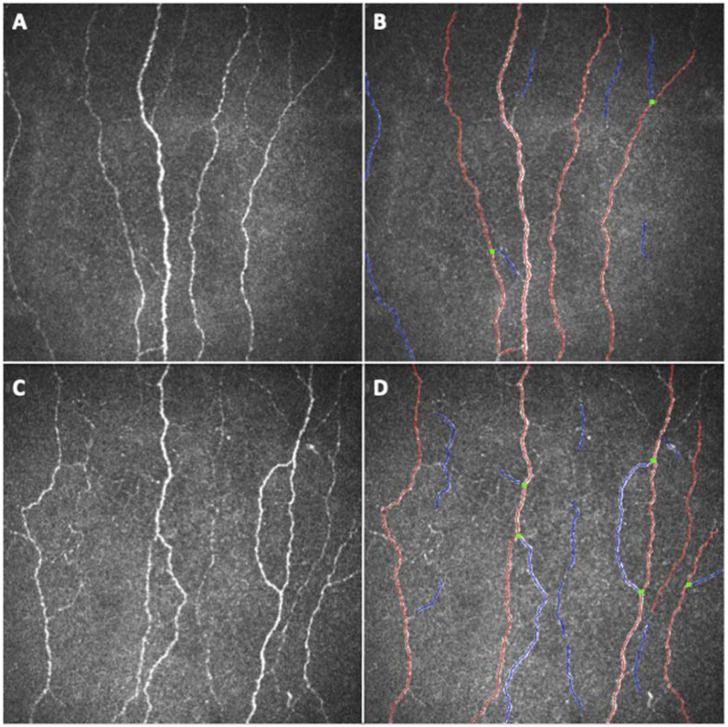
In-vivo corneal confocal microscopy images of the sub-basal nerve plexus of (**A**) a healthy subject and (**C**) a patient recovered from coronavirus disease 2019 (COVID-19) and their respective ACCMetrics analysis images (**B**,**D**), showing the main fibers in red, the branched fibers in blue and the branching points in green. (**B**) The ACCMetrics analysis of (**A**) compared to (**D**) the ACCMetrics analysis of (**C**) shows an increased number of branching points.

**Figure 2:**
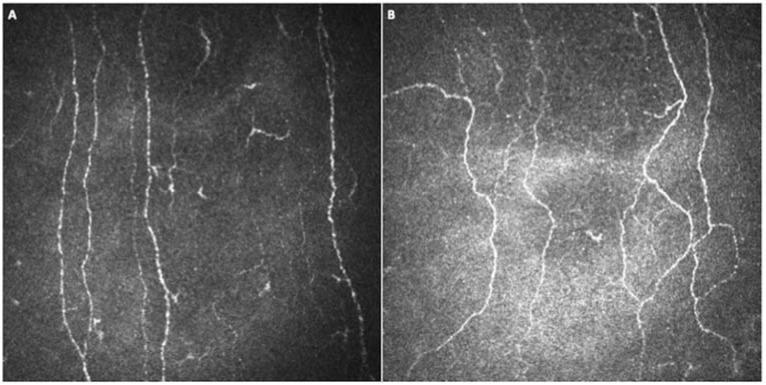
In-vivo corneal confocal microscopy images of the sub-basal nerve plexus of (**A**) a healthy subject and of (**B**) a patient recovered from coronavirus disease 2019 (COVID-19), showing (**B**) more tortuous corneal fibers in the patient’s examination compared to control.

CNBD and FT were significantly higher (*p* = 0.0131 and *p* < 0.0001, respectively) and CTBD was higher, with a borderline significance, (*p* = 0.0556) in the COVID-19 group compared to controls (Figure 1 and Figure 2).

**Figure 3:**
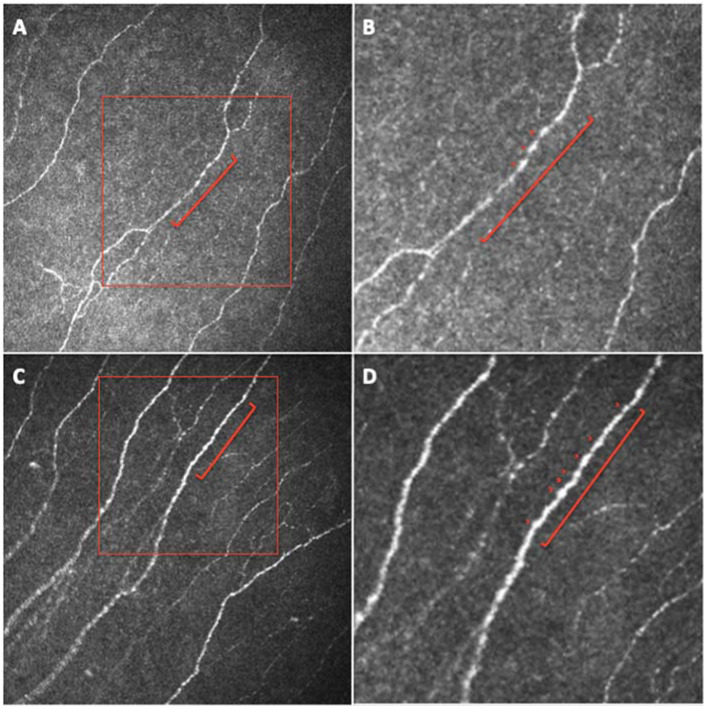
In-vivo corneal confocal microscopy images of the sub-basal nerve plexus of (**A**,**B**) a patient recovered from coronavirus disease 2019 (COVID-19) and (**C**,**D**) a healthy subject. Red lines in (**A**,**C**) indicate 100 microns of fiber; (**B**,**D**): magnification of red boxes in (**A**,**C**), respectively. Red arrowheads indicate corneal nerve beadings.

CNFW and NBe were significantly lower in patients compared to healthy controls (*p* = 0.0056 and *p* = 0.0045, respectively) (Figure 3).

There was no significative difference between the two groups regarding the other corneal sub-basal plexus parameters, nor between DC density (Table 2). Moreover, the regression model comparing all CCM parameters and time since recovery showed that only CNBD (*p* = 0.0289) and CTBD (*p* = 0.0169) parameters seemed to have a positive correlation with time since recovery.

Each CCM parameter was also compared among COVID-19 patients with different disease severity and different therapies during the course of their disease. The following (previously defined) parameters and therapies were considered and *p*-value results are reported in Table 3: duration of positivity, hospitalization, and length of hospitalization, admission to intensive care, need for oxygen therapy, mechanical ventilation, intubation and pharmacological therapy, including: corticosteroids, NSAID, paracetamol, antibiotics, antiviral drugs, hydroxychloroquine and/or chloroquine, heparin and/or other anticoagulants, biological drugs, plasma, and glutathione.

Any influence secondary to disease severity or pharmacologic therapy on the significant corneal findings in the COVID-19 group (Table 2) was then studied using multiple linear regression analysis, also adjusted for systemic comorbidities as potential confounders (Table 4). The variables chosen to be tested in the multiple linear regression analysis were those which resulted statistically significant (*p* < 0.10) or with borderline significance for at least one of the corneal parameters in univariate analysis (Table 3).

The resulting multiple linear regression analysis showed that CNBD and CTBD were significantly increased in patients taking antiviral drugs (26.8 ± 17.7 vs. 22.9 ± 20.0, *p* = 0.0207; 46.9 ± 28.4 vs. 39.2 ± 28.0, *p* = 0.0050 respectively) while significantly decreased in those receiving corticosteroids (21.7 ± 15.6 vs. 28.0 ± 22.6, *p* = 0.0359; 38.2 ± 47.4, *p* = 0.0462). FT was positively influenced by antiviral (3.09 ± 0.84 vs. 2.87 ± 0.91, *p* = 0.0332) and anticoagulant drugs (3.06 ± 0.84 vs. 2.71 ± 0.98, *p* = 0.0054) and negatively influenced by corticosteroids (2.93 ± 0.90 vs. 3.03 ± 0.88, *p* = 0.0520) (Table 4).

The relationship between the significantly altered corneal sub-basal nerve plexus findings in COVID-19 groups (CNBD, CTBD, CNFW, FT, and NBe) and symptoms showed only a sporadic correlation between CNBD and language disorders (*p* = 0.0080), CNFW and headache (*p* = 0.0450), and NBe and gastrointestinal symptoms (*p* = 0.0272) (Table 5).

## 4. Discussion

Clinical manifestations of COVID-19 may widely vary from no symptoms to multiple organ failure [7]. This infectious disease has been firstly and mainly reported as a respiratory syndrome with fever, fatigue, dyspnea, dry cough, myalgia, and pneumonic infiltrates in both lungs [7,24]. However, COVID-19 has proven to be a multiorgan disease and several neurological manifestations have been reported, including acute encephalitis, cerebrovascular diseases, and peripheral neuropathy [2]. Anosmia and ageusia are common and early findings in COVID-19. Loss of chemical sensations may also be associated, as a consequence of the nociceptive sensory neurons-mediated effect. Headache and neuropathic pain are also common manifestations [6]. Moreover, several post-infectious complications of COVID-19 affecting the brain or peripheral nerve fibers have been reported [24]. Dyspnea, joint pain, chest pain, and cough may also persist after recovery, and they are mediated, at least in part, by nociceptors [6].

The nerve fibers of the human cornea are nociceptive Aδ and C fibers [8]. In the corneal stroma, nerves organize in parallel to collagen lamellae, branch into smaller fascicles as they proceed toward the superficial stroma, and form interconnections to create the anterior plexuses. Along their course, these long nerve bundles divide into numerous smaller branches that connect to each other, giving rise to a delicate nerve network: the sub-basal nerve plexus, which innervates the corneal epithelium [8]. Small fibers neuropathy (SFN) is characterized by structural abnormalities of small nerve fibers (myelinated Aδ and unmyelinated C) with the degeneration of the distal terminations of nerve endings. SFN is characterized by the development of sensory and autonomic dysfunction, significantly affecting patients’ quality of life. The most common cause of SFN is type 2 diabetes mellitus and glucose intolerance. However, elevated erythrocyte sedimentation rate and C-reactive protein levels, reduced complement, and markers of autoimmune disorders are also common in SFN patients. Indeed, SFN may occur in immune-mediated disorders and infectious diseases [5]. Recently, the relevance of small fiber involvement in COVID-19 has been noted, since sensory small fibers may play a relevant role in the response to SARS-CoV-2-induced damage in the airway pathways and in the regulation of gastrointestinal motility [4].

Clinical diagnosis of SFN usually requires confirmation by skin biopsy, but recently non-invasive methods have been proposed to assess small fiber damage, of which the most important and validated is corneal confocal microscopy [5]. CCM is a non-invasive diagnostic modality to visualize and quantify the small corneal fibers originally derived from the first branch of the trigeminal nerve [11,12]. Qualitative and quantitative analysis of the finest corneal nerves, particularly the corneal sub-basal nerve plexus, may detect early and precisely small fibers diseases [9]. Therefore, the possibility of automatic and standardized evaluation criteria has made this technique suitable for diagnosing SFN in an objective manner. The diagnostic sensitivity and specificity of CCM in diabetic polyneuropathy, for example, are 91% and 93%, respectively, and such specificity has been proven to exactly correlate with results from skin biopsy [5].

The susceptibility of the ocular surface to SARS-CoV-2 has been already reported [25]. Conjunctival swab samples from the tears of infected individuals have proven positive for SARS-CoV-2 RNA, using RT-PCR, independently of ocular manifestations [7]. Nevertheless, the mechanisms of SARS-CoV-2-induced cellular damage remain unknown, particularly regarding the neural component, including the ocular and corneal one. Recently, corneal small nerve fiber loss has been reported in small cohorts of patients recovered from COVID-19 [22,26]. However, the clinical manifestations, the severity of the disease, and the required therapies may widely vary among patients, strongly influencing the post-infection clinical features and organ damage in different patients. In particular, a number of toxic causes have been reported to contribute to the development of SFN, from chemotherapy to antiretroviral therapy [5,11,27].

Using CCM, we found reduced fiber width and number of beadings, with increased branching and tortuosity of small fibers in the sub-basal nerve plexus of a large population of patients previously affected by COVID-19, compared to age-matched healthy subjects (Figure 1, Figure 2 and Figure 3). Therefore, the corneal sub-basal nerve plexus of patients recovered from COVID-19 shows significant fiber parameters changes, with thinner, suffering fibers devoid of nerve beadings. Nerve beadings, i.e., the accumulation of mitochondria along the nerve, represent the metabolic reservoir of the sub-basal nerve plexus, contributing to the maintenance of corneal integrity, and are not influenced by aging [13]. Therefore, the reduced number of nerve beadings reveals a pathologic metabolic activity of small nerve fibers, which also persists after clinical recovery from COVID-19. With regard to tortuosity, it has been shown to be increased in regenerating nerves, in sciatic nerve crush experiments, as well as in diabetic neuropathy and it is considered a morphologic marker of nerve degeneration and an attempt at fiber repair [9,21]. Accordingly, the increase in the number of branches may be related to a regenerative stimulus secondary to nerve damage. A similar phenomenon has been reported for axonal motor fibers in Duchenne muscular dystrophy [28]. However, the multivariate analysis showed that both nerve branching and tortuosity were influenced by antiviral drugs (increased) and corticosteroids (decreased), confirming the possible influence of therapies on some small fiber changes [5]. On the contrary, no therapeutic agents were shown to influence the reduction of fiber width and the number of beadings. Moreover, no systemic factors (including the need and length of hospitalization, the admission to intensive care, and oxygen therapy) and comorbidities (e.g., arterial hypertension) were found to influence nerve fiber changes in COVID-19 group. These data seem to suggest a direct susceptibility of small nerve fibers to SARS-CoV-2-induced damage, only partly influenced by antiviral and corticosteroid therapies, and independent of the severity of the systemic acute disease, at least in the recovery phase. Even in studies on diabetic patients, corneal sub-basal nerve plexus alterations were independent of glycemic control or disease duration [9,21]. The absence of a significant relationship between systemic factors and corneal nerve alterations detected in our population suggests a parallel and direct damage of small nerve fibers from the virus, not significantly influenced by the complex systemic mechanisms activated by the infection [29]. Surprisingly, we did not find a reduction also in CNFL and CNFD, as previously found in other neuropathic patients (such as diabetic patients) [30]. In our study, the fiber damage is expressed by the reduction in the COVID-19 group of NBe and CNFW, while the increase of CNBD and FT demonstrates a regenerative stimulus and an attempt at fiber repair. It would be interesting to evaluate with longitudinal studies the progression of fiber damage with regard to the different parameters.

Different hypotheses have been proposed for the pathogenesis of SARS-CoV-2 neurological involvement, mainly virus-induced hyperinflammatory and hypercoagulable state, direct virus infection, and post-infectious immune mediated processes [3]. The cytokine storm has been suggested as a possible cause of the damage to the afferent hypoxia-sensing neurons in people with COVID-19 [31]. Moreover, neuropathological studies have also shown the association of SARS-CoV-2 with microglial and lymphoid activation in neural tissues [32]. The possible immune-mediated component of SFN in post-COVID-19 syndrome may be relevant for the use of immunotherapy in the control of symptoms [24].

We did not find a significant difference in dendritic cell density in patients recovered from COVID-19 and healthy subjects. DCs density has been shown to be higher in eyes affected by immune-mediated inflammation and a possible interaction between DCs and nerves in the pathogenesis of neuropathy may be related to neuro-immune communication [23,33]. However, it has also been suggested that DCs and sensory nerve fibers/endings are intimately connected and functionally interdependent in the cornea and during epithelial wound healing, and corneal denervation has been associated with a reduction in DC density [20]. Therefore, our results in terms of DCs density may be due to the relatively long follow-up after the COVID19 in our population, which may have allowed the resolution of the local inflammatory milieu with the persistence of the nerve damage. However, it may also suggest a more direct virus-induced neural damage, compared to the inflammatory one. SARS-CoV-2 enters human host cells through coronavirus-associated receptors and factors (SCARFs), including cleaving transmembrane proteases angiotensin-converting enzyme 2 (ACE2) and transmembrane protease serine 2 (TMPRSS2). ACE2 and TMPRSS2 are expressed on the human ocular surface [4,33]. Shiers et al. have recently shown that angiotensin-converting enzyme 2 (ACE2) mRNA is expressed by a subset of nociceptors, suggesting that SARS-CoV-2 may gain access to the nervous system by entering into neurons that form free nerve endings in skin and other organs [6]. Moreover, the ACE2 not only serves as a critical determinant of SARS-CoV-2 transmissibility, but also regulates mitochondrial functions [25]. It regulates nicotinamide adenine dinucleotide phosphate (NAD(P)H) oxidase, leading to the increased production of reactive oxygen species in the mitochondria, which are mainly involved in energy production, but also in other functions, such as ion homeostasis, cellular signaling, differentiation, and cell death/survival [34]. Virus RNAs can also enter mitochondria, compromising mitochondrial integrity, as suggested in our population by the reduced number of NBe at CCM. The alteration of mitochondria by viruses such as SARS-CoV-2 deranges mitochondrial functions, leading to cell damage and enabling host defense evasion strategies [34]. A direct nerve invasion by the virus still needs to be investigated. However, this underlines the relevance of small fiber involvement in SARS-CoV-2 infection [4].

Our study confirms the damage to small fibers secondary to SARS-CoV-2 infection, even months after recovery, and independently of other systemic and therapeutic factors. Even if the exact pathogenetic mechanism inducing this complication needs to be clarified, CCM proved to be a useful non-invasive diagnostic tool to identify and quantify the involvement of fibers. The main limitation of this study is that patients had not underwent an extensive ocular examination but only selected procedures, in accordance with strict safety measures considered adequate to the study period (November 2020–January 2021). Another limitation of the study is that we do not have details about the COVID-19 variants of the studied patients. Moreover, they did not receive any quantitative neurological assessment during and after COVID-19. Our data did not show strong, clear, clinically significant relationships between CCM parameters and systemic COVID-19-related symptoms, including the neurological ones (cognitive impairment, memory disorders, ageusia/anosmia, ocular pain, etc., except for a single significant value, between CNBD and language disorders). However, no electrophysiologic test or skin biopsy was performed, and therefore we could not correlate the corneal nervous involvement with the systemic one. Recently, Bitirgen et al. reported more severe small fiber damage, detected at CCM, in patients with neurological symptoms (defined using questionnaires) four weeks after acute COVID-19 [22]. Neuropathy was also not assessed using objective measures (quantitative sensory testing, nerve conduction studies, skin biopsy) in Bitirgen’s study [22]. However, CCM has proven to correlate well with other objective methods of peripheral neuropathy evaluation [5]. Therefore, future longitudinal studies may not only clarify the evolution of SARS-CoV-2 related small fiber damage, but also a definitive role of CCM in the early and non-invasive detection of peripheral nervous system involvement in COVID-19.

## 5. Conclusions

Our study showed that SARS-CoV-2 infection may induce significant morphological changes in small peripheral nerve fibers as documented by CCM, regardless of systemic conditions and therapy. These quantitative data demonstrate that patients recovered from COVID 19 are still affected by small fiber peripheral neuropathy, similarly to other neuronal degenerative diseases. Even if the exact pathogenetic mechanism is still unclear, this aspect should be considered in the follow-up of patients affected by COVID-19. Moreover, CCM may also be a useful non-invasive diagnostic tool to identify and monitor small fiber neuropathy in COVID-19.

## Figures and Tables

**Table 1:** Demographic and clinical characteristics of studied subjects.

		COVID-19 Group (*n* = 151)	Control Group (*n* = 46)
Demographic features			
Male/female, *n* (%)		83 (55%)/68 (45%)	16 (34.8%)/30 (65.2%)
Mean age, y ± SD		56.8 ± 14.2	49.4 ± 26.5
Clinical features			
Mean time of positivity, days ± SD		19.3 ± 10.5	
Mean time from recovery, days ± SD		144.4 ± 104	
COVID-19 severity parameters			
Hospitalization, *n* (%)		135 (89.4%)	
Time of hospitalization, days ± SD		13 ± 2.6	
	Intensive care management *n* (% of hospitalized)	30(22.2%)	
	Oxygen therapy *n* (% of hospitalized)	90 (66.7%)	
	Mechanical ventilation *n* (% of hospitalized)	16 (11.9%)	
	Intubation *n* (% of hospitalized)	7 (5.2%)	
Symptoms, *n* (%)			
Fever		137 (90.7%)	
Gastrointestinal symptoms		55 (36.4%)	
Respiratory symptoms		122 (80.8%)	
Osteoarticular symptoms		68 (45.0%)	
Ageusia/anosmia		104 (68.9%)	
Perception of reduction in visual acuity		35 (23.2%)	
Ocular pain		8 (5.3%)	
Redness and/or burning eye sensation		19 (12.6%)	
Dizziness		36 (23.8%)	
Headache		69 (45.7%)	
Syncope/fainting		18 (11.9%)	
Language disorders		20 (13.2%)	
Cognitive impairment		56 (37.1%)	
Memory disorders		49 (32.5%)	
Comorbidities, *n* (%)			
Arterial hypertension		47 (31.1%)	
Previous cardiovascular events		11 (7.3%)	
Previous chemotherapy treatment		1 (0.7%)	
Pharmacologic therapy, *n* (%)			
Corticosteroids		85 (56.3%)	
NSAID		5 (3.3%)	
	Ketoprofen lysine salt *n* (% of NSAID)	1 (20%)	
	Diclofenac *n* (% of NSAID)	1 (20%)	
	Ketorolac tromethamine *n* (% of NSAID)	3 (60%)	
Paracetamol		116 (76.8%)	
Antibiotics		125 (82.8%)	
Antiviral drugs		59 (39.1%)	
	Lopinavir/Ritonavir *n* (% of antiviral drugs)	24 (40.7%)	
	Remdesivir *n* (% of antiviral drugs)	33 (55.9%)	
	Lopinavir/Ritonavir + Remdesivir *n* (% of antiviral drugs)	2 (3.4%)	
Hydroxychloroquine and/or Chloroquine		63 (41.7%)	
Heparin and/or other anticoagulants		115 (76.2%)	
Biological drugs (Tocilizumab)		10 (6.6%)	
Plasma		21 (13.9%)	
Glutathione		17 (11.3%)	
None		3 (2%)	

COVID-19: Coronavirus disease 2019; SD: standard deviation; NSAID: non-steroidal anti-inflammatory drugs.

**Table 2:** Corneal confocal microscopy data in COVID-19 and control groups.

Corneal Parameters	COVID-19 Group Mean ± SD	Control Group Mean ± SD	*p*-Value
CNFD (*n*/mm^2^)	17.2 ± 8	15.3 ± 7.9	0.0911
CNBD (*n*/mm^2^)	24.4 ± 19.2	17.7 ± 15.7	**0.0131**
CNFL (mm/mm^2^)	11.9 ± 4.0	11.3 ± 3.6	0.2737
CTBD (*n*/mm^2^)	42.2 ± 28.4	34.7 ± 20.8	**0.0556**
CNFA (mm^2^/mm^2^)	0.006 ± 0.002	0.005 ± 0.002	0.6670
CNFW (mm/mm^2^)	0.022 ± 0.002	0.023 ± 0.002	**0.0056**
FT (range 0–4)	3.0 ± 0.9	2.2 ± 0.9	**<0.0001**
NBe (*n*/100 µm)	9.7 ± 2.5	10.7 ± 3.0	**0.0045**
DC density (cells/mm^2^)	34.4 ± 56.5	37.9 ± 64.5	0.6521

COVID-19: Coronavirus disease 2019; SD: standard deviation; no: number; CNFD: corneal nerve fiber density; CNBD: corneal nerve branch density CNFL: corneal nerve fiber length; CTBD: corneal nerve total branch density; CNFA: corneal nerve fiber area; CNFW: corneal nerve fiber width; FT: fiber tortuosity; NBe: number of beadings; DC: dendritic cells; *n*: number; mm: millimeter; significant or borderline results (level of significance 0.05) in bold.

**Table 3:** Corneal confocal microscopy data in COVID-19 patients, according to disease severity and therapies (*p*-values from univariate analyses).

	CNFD	CNBD	CNFL	CTBD	CNFA	CNFW	FT	NBe	DC Density
Severity parameters									
Time of positivity (≤18 vs. >18 days)	0.7397	0.9243	0.8344	0.8898	0.5183	0.9447	0.6245	0.3923	0.3666
Hospitalization	0.2673	**0.0944**	**0.0758**	**0.0864**	**0.0288**	0.3680	0.6907	0.2067	0.9874
Time of hospitalization (≤10 vs. >10 days)	0.2165	0.2678	0.2405	0.3217	**0.0915**	0.8755	**0.0156**	0.7971	0.2347
Intensive Care	0.4689	0.8935	0.7554	0.9846	0.7886	0.8678	**0.0247**	0.5724	0.2429
Oxygen therapy	0.5065	0.4091	0.4631	0.2800	**0.0652**	0.6255	0.1774	0.9998	**0.0022**
Mechanical ventilation	0.9932	0.7857	0.6957	0.8314	0.5068	0.9970	0.2433	0.5354	0.4255
Intubation	0.5148	0.4012	0.9176	0.7403	0.9447	0.4862	0.1924	0.5202	0.5414
Pharmacological treatment									
Corticosteroids	0.3447	**0.0226**	0.1261	**0.0264**	**0.0127**	0.5539	0.4399	0.5285	0.1798
NSAID	0.5631	0.6298	0.3563	0.8720	0.4837	0.9981	0.8160	0.3321	0.3753
Paracetamol	0.7727	0.3876	0.7303	0.2310	0.4858	**0.1094** **B**	0.5737	0.2188	0.1296
Antiobiotics	0.8307	0.9566	0.8802	0.6939	0.9676	0.1302	0.4049	0.6755	0.3946
Antiviral drugs	0.2408	0.1725	0.2256	**0.0644**	0.3044	0.3102	**0.0360**	0.2418	0.8109
Hydroxychloroquine and/or chloroquine	0.1575	**0.0375**	**0.1096** **B**	**0.0125**	0.1930	0.3454	0.1244	0.9260	0.8385
Heparin and/or other anticoagulants	0.4995	0.2481	0.3710	0.4326	0.7645	0.9129	**0.0130**	0.8741	**0.0202**
Biological drugs	0.2126	0.1624	0.1938	0.2644	0.2220	0.1167	0.3298	0.9717	0.4412
Plasma	0.2834	**0.1094** **B**	0.1214	**0.1007** **B**	0.3212	0.5796	0.5279	0.5747	0.3951
Glutathione	0.4187	0.7139	0.7005	0.6345	0.8766	0.5351	0.8353	0.4833	0.8479

CNFD: corneal nerve fiber density; CNBD: corneal nerve branch density CNFL: corneal nerve fiber length; CTBD: corneal nerve total branch density; CNFA: corneal nerve fiber area; CNFW: corneal nerve fiber width; FT: fiber tortuosity; NBe: number of beadings; DC: dendritic cells; significant results (level of significance 0.10) in bold; B: borderline significance.

**Table 4:** Relationship between CCM corneal and systemic clinical data in COVID-19 group patients (*p*-values from multiple regression analysis).

	CNBD	CTBD	CNFW	FT	NBe
Severity parameters					
Hospitalization	0.2889	0.1979	0.2219	0.0851	0.1577
Time of hospitalization	0.2981	0.2437	0.9785	0.0848	0.8486
Intensive Care	0.4724	0.3189	0.9650	0.4895	0.6464
Oxygen treatment	0.3062	0.1324	0.6790	0.6549	0.4861
Treatments					
Corticosteroids	**0.0359**	**0.0462**	0.3872	**0.0520**	0.5518
Paracetamol	0.3304	0.1849	0.0879	0.4943	0.2429
Antiviral drugs	**0.0207**	**0.0050**	0.1909	**0.0332**	0.4378
Hydroxychloroquine and/or chloroquine	0.6489	0.3885	0.3436	0.7389	0.9643
Heparin and/or other anticoagulants	0.3908	0.6952	0.8420	**0.0054**	0.7310
Plasma	0.4862	0.5878	0.1823	0.4281	0.9789
Comorbidities					
Hypertension	0.7650	0.9929	0.9242	0.9450	0.4609
Previous cardiovascular events	0.3452	0.3535	0.5109	0.9079	0.3483
Previous chemotherapy	0.7252	0.6377	0.7692	0.6234	0.6092

CCM: corneal confocal microscopy; COVID-19: Coronavirus disease 2019; CNBD: corneal nerve branch density; CTBD: corneal nerve total branch density; CNFW: corneal nerve fiber width; FT: fiber tortuosity; NBe: number of beadings; significant and borderline results (level of significance 0.05) in bold.

**Table 5:** Relationship between CCM corneal data and symptoms (*p*-values from univariate analyses).

Symptoms	CNBD	CTBD	CNFW	FT	NBe
Fever	0.4125	0.2350	0.8786	0.2128	0.2119
Gastrointestinal symptoms	0.0840	0.0791	0.6218	0.3945	**0.0272**
Respiratory symptoms	0.3148	0.1493	0.2841	0.9281	0.0680
Osteoarticular symptoms	0.5269	0.6639	0.3984	0.2361	0.5874
Ageusia/anosmia	0.9016	0.5748	0.5248	0.9234	0.9253
Perception of reduced visual acuity	0.1765	0.1154	0.0666	0.3943	0.6027
Ocular pain	0.2141	0.2799	0.3539	0.1071	0.0782
Redness/burning eye sensation	0.1860	0.1340	0.1425	0.3164	0.3209
Dizziness	0.6349	0.3248	0.0970	0.5814	0.8013
Headache	0.4860	0.5950	**0.0450**	0.0660	0.3426
Syncope/fainting	0.6527	0.8640	0.0664	0.6352	0.6766
Language disorders	**0.0080**	0.0599	0.4924	0.0520	0.6023
Cognitive impairment	0.7599	0.9652	0.6435	0.4055	0.5293
Memory disorders	0.1174	0.2619	0.5597	0.8830	0.8032

CCM: corneal confocal microscopy; CNBD: corneal nerve branch density; CTBD: corneal nerve total branch density; CNFW: corneal nerve fiber width; FT: fiber tortuosity; NBe: number of beadings; significant results (level of significance 0.05) in bold.

## Data Availability

The data presented in this study are available in the Article. Eventual additional data are available on request from the corresponding author.
